# To Kill the Calyces: A Rare Case of Xanthogranulomatous Pyelonephritis Complicated by Emphysematous Pyelonephritis

**DOI:** 10.1155/crra/7650184

**Published:** 2025-11-21

**Authors:** Brandon Simons, Aaron Geril, Amir Hedayati

**Affiliations:** Department of Radiology, University of Central Florida College of Medicine, Orlando, Florida, USA

## Abstract

Xanthogranulomatous pyelonephritis (XPN) is a rare chronic renal infection associated with obstructive nephrolithiasis and recurrent urinary tract infections. Emphysematous pyelonephritis (EPN) is an even rarer necrotizing infection characterized by intrarenal gas, most commonly seen in patients with uncontrolled diabetes mellitus. Concurrent presentation of XPN and EPN is exceedingly uncommon, with only a few cases reported. We present a case of a 61-year-old female with no history of diabetes who presented with left flank pain, systemic symptoms, and laboratory evidence of infection and microcytic anemia. Abdominal CT revealed imaging features consistent with XPN—including hydronephrosis, a staghorn calculus, and the classic “bear paw” sign—alongside intrarenal gas, consistent with EPN. Urine culture grew *Proteus mirabilis*. The patient underwent nephrectomy and recovered uneventfully with antimicrobial therapy. This case is notable for the rare co-occurrence of XPN and EPN in a euglycemic patient, supporting the hypothesis that chronic obstruction and infection may be sufficient to induce gas-forming infections, even in the absence of hyperglycemia. Awareness of atypical presentations is essential, as early recognition and surgical management are critical to reducing morbidity in these severe renal infections.

## 1. Introduction

Xanthogranulomatous pyelonephritis (XPN) is an uncommon form of chronic pyelonephritis, most often secondary to nephrolithiasis with hydronephrosis. XPN typically occurs in the setting of recurrent urinary tract infections in middle-aged women and presents with fever and flank pain [1]. The most common pathogens isolated in urine cultures are *Escherichia coli*, *Proteus mirabilis*, and *Pseudomonas* [2]. XPN is diagnosed based on imaging findings and pathology. CT scan often reveals a unilateral “bear paw sign” comprised of multiloculated hypodense lobules surrounded by a rim of enhancement along with kidney stones or calculi [3]. Pathology grossly demonstrates yellow necrosis comprised of lipid-laden macrophages with peripheral layers of orange-colored tissue. Multiple layers of inflammatory cells concentrically surrounding a calyx are seen on microscopic evaluation [4].

Emphysematous pyelonephritis (EPN) is a severe, potentially life-threatening, necrotizing infection resulting in gas in the renal parenchyma, collecting system, or perinephric tissue. It is commonly due to uncontrolled diabetes mellitus with or without ureteral obstruction and is more often found in females. The causative pathogens are similar to XPN [5]. EPN may be complicated by acute kidney injury, septic shock, and bacteremia [6]. Intrarenal gas on ultrasound or CT supports the diagnosis [7].

Herein, we report a case of concurrent XPN and EPN. Rarely do these two pathologies occur together.

## 2. Case Presentation

A 61-year-old female with a past medical history of nicotine dependence presented to the emergency department with 2 weeks of worsening left flank pain associated with subjective fever, nausea, vomiting, dysuria, and decreased appetite. The patient also endorsed fatigue, dyspnea on exertion, and pica but denied hematemesis, hematochezia, melena, hematuria, or other sources of bleeding. The patient stated she was postmenopausal, and her last menstrual cycle was about 10 years ago. She had not seen a primary care physician in about 40 years and denied prior hospitalizations or surgeries. She did not take over-the-counter or prescribed medication other than recently starting BC Powder three to five times per day the week prior for her flank pain.

On presentation to the emergency department, the patient was afebrile (98.2°F), saturating 97% O_2_ on room air, normotensive (132/81 mm Hg), with a normal respiratory rate (18 breaths/min), and had a pulse of 126 beats/min. On physical examination she was ill-appearing and uncomfortable. Abdominal examination revealed diffuse tenderness to palpation without guarding or rebound and positive left costovertebral angle tenderness. The remainder of the physical examination was unremarkable.

Her labs upon admission revealed mild leukocytosis of 11.9 K/*μ*L (4.0–10.5 K/*μ*L) with neutrophilia of 79.0% (34.0%–71.1%), mild hyponatremia of 132 mEq/L (135–145 mEq/L), mild hypochloremia of 96 mEq/L (98–107 mEq/L), elevated creatinine of 1.31 mg/dL (0.6–1.3 mg/dL), estimated GFR of 46, normal glucose of 107 mg/dL, microcytic anemia (hemoglobin of 7.2 g/dL) (11.2–15.7g/dL) and MCV of 75 fL (79.4–94.8 fL), and thrombocytosis of 783 K/*μ*L (150–400 K/*μ*L). Urinalysis revealed large blood (51–100 urine RBCs/HPF), large leukocyte esterase (> 100 urine WBCs/HPF), proteinuria (> 300 urine protein), negative nitrites, and 5+ urine bacteria.

**Figure 1:**
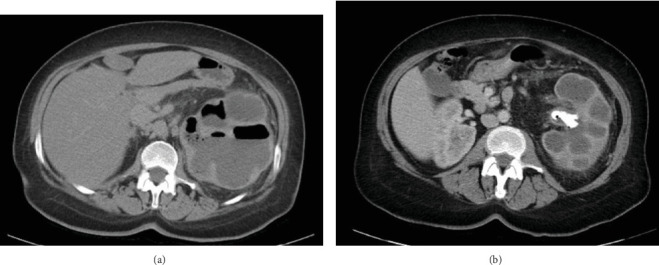
CT abdomen and pelvis with contrast axial views demonstrating severe hydronephrosis, dilated calices, and thin renal parenchyma of the left kidney. (a) Gas within the left renal collecting system in the calices. (b) Large stone within the left renal pelvis measuring 3.3 cm.

**Figure 2:**
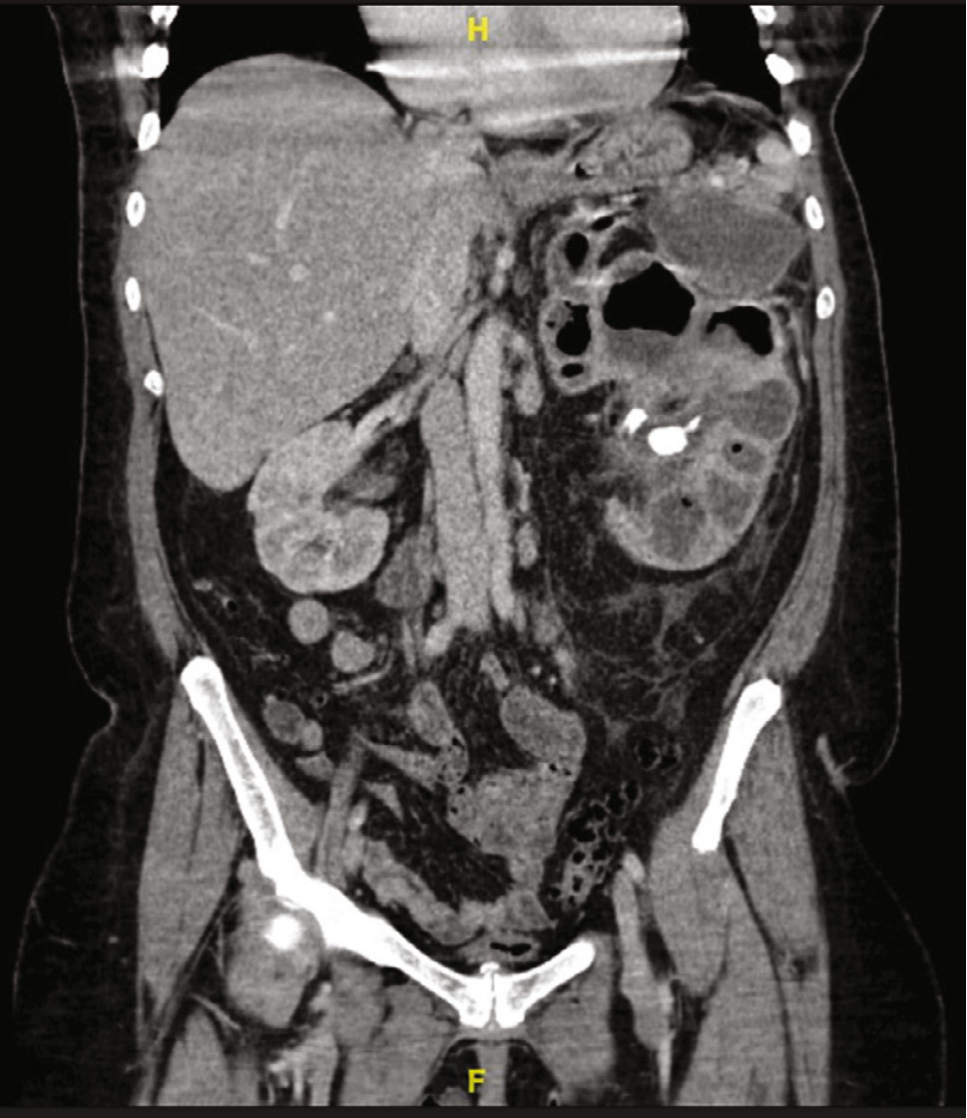
CT abdomen and pelvis with contrast coronal view depicts the characteristic “bear paw” sign seen in xanthogranulomatous pyelonephritis.

An initial chest x-ray demonstrated no acute cardiopulmonary process. CT abdomen and pelvis revealed severe hydronephrosis and dilated calices of the left kidney with a 3.3 cm stone within the left renal pelvis along with stones in the lower poles of the left kidney. There was also gas within the left renal collecting system in the calices (Figures 1 and 2). The findings were concerning for XPN with emphysematous. The patient was transferred to inpatient care and treated with ceftriaxone 2.0 g IV q 24 h.

Nephrectomy was initially delayed due to the need for medical stabilization and repeat blood transfusions. Once stabilized, the patient underwent transabdominal left radial nephrectomy without complication. Grossly, pathology revealed severe acute and chronic pyelonephritis with multiple intraparenchymal abscesses and calculi within the renal pelvis. No malignancy was identified. The patient significantly improved and was discharged with a 7-day course of oral cefdinir.

## 3. Discussion

XPN and EPN are uncommon causes of pyelonephritis. The incidence of XPN is 0.6%–1.0% of all renal infections, while the occurrence of EPN is even rarer [8]. To our knowledge, only 11 previous cases of concurrent XPN and EPN have been reported.  Table 1 outlines the clinical features of the combined XPN and EPN cases. Additionally, Alshyarba describes a metachronous case of XPN and EPN affecting contralateral kidneys [17]. XPN and EPN most commonly involve patients between 40 and 60 years old. Females are disproportionately affected, most likely due to the increased occurrence of urinary tract infections [8]. The mean age of patients with combined XPN and EPN in Table 1 is 53.3 years old (*σ* = 13.1), and 10 out of 12 cases were female.

XPN often arises due to obstruction and infection from underlying nephrolithiasis resulting in a chronic, yet incomplete, inflammatory process due to defective macrophagic processing of bacteria. A xanthogranulomatous kidney arises from recurrent infections and inflammation resulting in the formation of granulomatous tissue composed of lipid-laden macrophages [18]. In severe, untreated cases, the infection may spread to the retroperitoneum or nearby organs [19]. EPN is a severe, necrotizing infection that arises in the renal parenchyma. Diabetes mellitus is the most common risk factor, which may be found in up to 95% of cases, while urinary tract obstruction occurs in 25%–40% of cases. Proposed requirements for developing EPN include the presence of gas-forming bacteria, high local tissue glucose levels, and impaired renal tissue perfusion [20]. The leading theory is uncontrolled diabetes mellitus causes impaired arterial perfusion promoting local anaerobic metabolism. High glucose levels foster a conducive environment for the growth of facultative anaerobic bacteria, such as *E. coli*, which produce gas through the fermentation of glucose and lactate. Nephrolithiasis acts as a nidus for urinary tract infections and subsequent pyelonephritis [20]. Overall, 10 out of 12 cases of combined XPN and EPN had a prior medical history of diabetes mellitus or presented with uncontrolled glucose levels. However, our case did not have a history of diabetes mellitus, and the patient presented with a normal random glucose of 107 mg/dL. This suggests that a xanthogranulomatous kidney may serve as a precursor to the formation of EPN. XPN likely resulted in septic infarction of renal tissues, creating a suitable anaerobic environment for gas-producing bacteria.

A recent systematic review of over 1000 cases found XPN to have a mortality rate of 1.4%, lower than previously reported, while EPN has an estimated mortality rate of 13%–25% [21, 22]. One death is documented in the cases of combined EPN and XPN from an intraoperative mycotic aneurysm rupture of the splenic artery during nephrectomy [13]. As in our case, treatment involves antibiotics with subsequent nephrectomy. Wen and Chen (Table 1) describe a case treated with percutaneous needle aspiration and antibiotics, due to patient refusal of nephrectomy, with a successful outcome [9].

## 4. Conclusion

This case presents the rare presentation of a 61-year-old female with combined XPN and EPN secondary to *P. mirabilis* nephrolithiasis. Unlike most other cases of simultaneous XPN and EPN, this patient did not have a history of diabetes mellitus or uncontrolled hyperglycemia. The patient's lack of previous medical care likely contributed to the development of XPN from a renal pelvic stone. The presence of EPN is likely a complication caused by the rapid deterioration of renal function from untreated XPN.

## Figures and Tables

**Table 1:** Characteristics of combined XPN and EPN cases. Adopted and updated from Wen and Chen [9].

**Author**	**Age**	**Sex**	**Diabetes**	**Renal history**	**Fever**	**Organism**	**Onset**	**Treatment**	**Outcome**
Langdale et al. [5]	28	F	Hyperglycemia	None	Yes	Mixed	2 weeks	Antibiotics, nephrectomy, and percutaneous drainage	Uneventful
Moríya et al. [10]	49	F	N/A	N/A	Yes	N/A	N/A	Antibiotics and nephrectomy	N/A
Chuang et al. (two cases) [1]	N/A	N/A	Yes (both)	N/A	N/A	*E. coli* (both)	N/A	Antibiotics and nephrectomy (both)	N/A
Punekar et al. [11]	70	F	Yes	N/A	Yes	*Klebsiella pneumoniae*	1 week	Antibiotics and nephrectomy	Uneventful
Ishigami et al. [12]	42	F	Hyperglycemia	N/A	No	*E. coli* and *Proteus*	2 weeks	Antibiotics, nephrectomy, and debridement	Uneventful
Wen and Chen [9]	78	F	Hyperglycemia	ESRD on hemodialysis and multiple UTIs	No	*Klebsiella pneumoniae*	1 week	Antibiotics and percutaneous needle aspiration	Uneventful
Edigin and Patel [13]	57	F	No	Prior pyelonephritis	Yes	*E. coli* and *Pseudomonas aeruginosa*	1 week	Antibiotics, nephrectomy, and percutaneous drainage	Death
Maghsoudi et al. [14]	30	M	No	Prior nephrolithiasis	Yes	N/A	N/A	Antibiotics and nephrectomy	N/A
Moudi et al. [15]	55	F	Yes	N/A	No	*E. coli* and *Klebsiella oxytoca*	N/A	Antibiotics and nephrectomy	Uneventful
Mhmed Ali and Haidar [16]	63	M	Yes	Prior nephrolithiasis	Yes	*Klebsiella*	2 months	Antibiotics and nephrectomy	N/A
Current case	61	F	No	None	No	*Proteus mirabilis*	2 weeks	Antibiotics and nephrectomy	Uneventful

Abbreviations: F, female; M, male; N/A, not available.

## Data Availability

The data that support the findings of this study are available from the corresponding author upon reasonable request.
